# Use of NCCN distress thermometer in cancer genetics patients

**DOI:** 10.1007/s00520-025-09473-y

**Published:** 2025-04-26

**Authors:** Aidan M. Kennedy, Andrea M. Murad, Erika S. Koeppe, Michelle B. Riba, Elena M. Stoffel, Michelle F. Jacobs

**Affiliations:** 1 Rogel Cancer Center, University of Michigan, 300 N. Ingalls, SPC 5419, Ann Arbor, MI 48109 USA; 2 Department of Internal Medicine, University of Michigan, 300 N. Ingalls, SPC 5419, Ann Arbor, MI 48109 USA; 3 Department of Psychiatry, University of Michigan, Ann Arbor, MI 48109 USA

**Keywords:** Hereditary cancer, Genetic counseling, Psychosocial assessment, Distress measures

## Abstract

**Purpose:**

Distress is not routinely measured in patients undergoing cancer genetic counseling. We evaluated the use of the National Comprehensive Cancer Network® (NCCN®) Distress Thermometer (DT) and Problem List (PL) in an unselected population of individuals presenting to a cancer genetics clinic to determine their utility in assessing distress in this population.

**Methods:**

Patients presenting to one cancer genetics clinic between April 2019 and March 2020 were asked to complete the DT and PL (2019 version). New patients aged 18 or older were divided into categories of low (DT score < 4) and high distress (DT score ≥ 4), consistent with NCCN groupings for evaluation of distress. Correlates of distress were explored using Chi-square and *t* tests.

**Results:**

Younger age and female sex assigned at birth were associated with reporting high distress. Personal history of cancer was not associated with high distress. Worry, fatigue, and sleep disturbance were the most commonly patient-reported problems.

**Conclusion:**

Findings from this study show that the DT can assist in identifying patients undergoing cancer genetic counseling who are experiencing high distress; however, research to develop a PL more specific to the concerns of the oncology genetic counseling patient population may be considered to better identify relevant patient problems.

**Supplementary Information:**

The online version contains supplementary material available at 10.1007/s00520-025-09473-y.

## Background

Explorations into the psychosocial experiences of patients in oncology settings have suggested these patients experience more distress than the general population [1]. To assess degree of distress, the National Comprehensive Cancer Network® (NCCN) developed the Distress Thermometer (DT), which prompts patients to report distress on a Likert scale ranging from zero (no distress) to ten (extreme distress) [2]. The DT is accompanied by a Problem List (PL) examining specific areas of distress, including physical, emotional, and practical concerns. The DT and PL have been used in patients with an active diagnosis of cancer, with 25–60% of patients with cancer reporting distress compared to 7% of the general population [3]. Common concerns reported by oncology patients include worry, anxiety, fears, sadness, and problems with housing, money, sleep, and memory [4, 5]. In one study, reported female gender and younger age were associated with higher reported distress [6]. There is an established relationship between higher levels of reported distress and negative oncology patient outcomes including dissatisfaction with care, poorer quality of life, and inferior survival rates [3].

Application of the DT to a patient population seeking care related to inherited cancer risk predisposition has been previously explored on a limited basis; 19–66% of individuals in this setting reported high distress, similar to rates reported in cancer patients [7–9]. Due to noted limitations in the PL in explaining differences in patient-reported distress in this population, one group created a 26-item Psychosocial Aspects of Hereditary Cancer (PAHC) questionnaire tailored to assess problems in patients presenting for cancer genetic counseling. To develop this tool, a literature review focused on articles related to “genetic counseling”, “cancer”, and “psychology” was performed and interpreted for relevant themes [9]. Insight was also gathered through interviews with care providers in the cancer genetics setting (including physicians, genetic counselors, social workers, and others), along with soliciting feedback from researchers active in psycho-oncology as well as genetic counseling patients themselves [9]. In benchmarking of this tool, however, writers noted challenges in determining consistent cutoff values across questions to balance positive predictive value with specificity [9]. In a following study comparing the consistency of the DT and the PAHC, while this newly developed questionnaire demonstrated limited correlation with reported distress, this was insufficient to adequately explain variance among DT responses [9]. Due to the lack of a single measure which both correlates with existing measures validated for assessing patient distress in the oncology setting, as well as specific to genetic counseling concerns, additional work is needed to determine if a different tool could be more effective in assessing concerns in this population. Here, we explored DT scores and items in the PL tool among unselected patients presenting to a cancer genetics clinic.

## Methods

All patients presenting to the Michigan Medicine Cancer Genetics Clinic between April 2019 and March 2020 were asked to complete the DT and PL (2019 version, Online Material 1) [2]. New patients aged 18 or older who completed the DT and PL were included in analysis. Demographic information including age, sex assigned at birth, race/ethnicity, personal history of cancer, and cancer history among first-degree relatives were obtained from clinic records. DT and PL responses were reviewed by the genetic counselor prior to initial contact and discussed with the patient at the genetic counselor’s discretion. Patients reporting high distress, endorsing issues from the PL, and/or with psychosocial concerns were offered a referral to psychological care services if indicated after discussion. There were no set criteria for when to discuss and/or place a referral to psychology. Consistent with the National Society of Genetic Counselors Code of Ethics, genetic counselors provided psychological support within their scope of practice and could offer a referral to an appropriately qualified psychology professional for a variety of reasons [10]. Reasons could include distress from a medical diagnosis, social stressors, patient request, complex family dynamics, adjustment to a new diagnosis, or other reasons. Those that declined were briefly questioned as to their reasons, but these were not formally tracked. Patients were divided into categories of low (DT score < 4) and high distress (DT score ≥ 4) for analysis, consistent with NCCN groupings for evaluation of distress [2]. Correlates of distress were explored using Chi-square and *t* tests.

## Results

Nine hundred and ninety-three patients presented to the clinic during the study period; 968 (97.5%) completed the DT and PL while 11 declined and 14 had incomplete responses. A further 301 were excluded as they were return patients (253) or were under the age of 18 (48), leaving 667 patients for analysis (Table 1). The study population overall had a mean DT score of 2.7 (standard deviation (SD) = 2.7); 218 (32.7%) were categorized as high distress (DT ≥ 4) (mean DT of 6.1, SD = 1.7).

**Table 1:** Participant characteristics

Characteristic	All subjects *n* = 667 (100.0%)	Subjects distress < 4 *n* = 449 (67.3%)	Subjects distress ≥ 4 *n* = 218 (32.7%)	*p* value difference between distress levels
Mean distress score (range, standard deviation)	2.7 (0–10, 2.7)	1.0 (0–3.5, 1.1)	6.1 (4–10, 1.7)	N/A
Mean number of distress items selected (range, standard deviation)	4.1 (0–39, 4.8)	2.4 (0–39, 3.5)	7.6 (0–25, 5.3)	< 0.00001
Mean age, years (range)	51.8 (18–88)	52.9 (18–87)	49.4 (19–88)	0.008
Female	393 (58.9%)	239 (53.3%)	154 (70.6%)	0.00003
Race/Ethnicity				
African American	26 (3.9%)	14 (3.1%)	12 (5.5%)	NS
Asian	11 (1.6%)	7 (1.6%)	4 (1.8%)	
Hispanic-NonWhite	4 (0.6%)	3 (0.7%)	1 (0.5%)	
Hispanic-White	7 (1.0%)	4 (0.9%)	3 (1.4%)	
Native American	2 (0.3%)	1 (0.2%)	1 (0.5%)	
White-NonHispanic	600 (90.0%)	411 (91.5%)	189 (86.7%)	
More than one race/other	10 (1.5%)	5 (1.1%)	5 (2.3%)	
Unknown	7 (1.0%	4 (0.9%)	3 (1.4%)	
Personal history cancer	340 (51.0%)	228 (50.8%)	112 (51.3%)	NS
Recent/current cancer (2018–2020)	201 (30.1%)	135 (30.1%)	66 (30.3%)	NS
Cancer in FDR	543 (81.4%)	363 (80.8%)	180 (82.3%)	NS
Cancer in 3 + FDR	126 (18.9%)	83 (18.5%)	43 (19.7%)	NS

*NS* not significant, *FDR* first-degree relative

Factors associated with being in the high distress group included younger age (mean of 49.4 versus 52.9 years; *p* = 0.008), female sex assigned at birth (70.6% versus 53.3%; *p* = 0.00003), and higher number of PL items selected (mean of 7.6 versus 2.4 *p* < 0.00001). No statistically significant differences were observed between reported degree of distress (low versus high) and race, having a personal history of cancer, recency of cancer diagnosis (within last 2 years versus more than 2 years ago), or family history of cancer. Participants reporting high distress most frequently endorsed worry, fatigue, nervousness, sleep, and pain as problems. Participants reporting low levels of distress most frequently endorsed fatigue, worry, sleep, pain, and tingling in the hands or feet as problems (Table 2).

**Table 2:** Frequency of reported problems divided by reported level of distress

All subjects, *n* = 667 (100.0%)				Subject distress < 4, *n* = 449 (67.3%)				Subjects distress ≥ 4, *n* = 218 (32.7%)			
Item	Rank	Count	%	Item	Rank	Count	%	Item	Rank	Count	%
Worry	1	215	32.2	Fatigue	1	88	19.6	Worry	1	136	62.4
Fatigue	2	200	30.0	Worry	2	79	17.6	Fatigue	2	112	51.4
Sleep	3	168	25.2	Sleep	3	75	16.7	Nervousness	3	93	42.7
Pain	4	145	21.7	Pain	4	63	14.0	Sleep	4	93	42.7
Nervousness	5	132	19.8	Tingling in hands/feet	5	56	12.5	Pain	5	82	37.6
Tingling in hands/feet	6	117	17.5	Skin dry/itchy	6	52	11.6	Sadness	6	76	34.9
Memory/concentration	7	111	16.6	Nose dry/congested	7	42	9.4	Depression	7	71	32.6
Sadness	8	108	16.2	Family health issues	8	40	8.9	Memory/concentration	8	71	32.6
Family health issues	9	102	15.3	Memory/concentration	9	40	8.9	Fears	9	64	29.4
Depression	10	96	14.4	Nervousness	10	39	8.7	Family health issues	10	62	28.4
Skin dry/itchy	11	96	14.4	Constipation	11	34	7.6	Tingling in hands/feet	11	61	28.0
Fears	12	93	13.9	Sadness	12	32	7.1	Work/school	12	55	25.2
Work/school	13	83	12.4	Nausea	13	30	6.7	Eating	13	45	20.6
Nose dry/congested	14	76	11.4	Fears	14	29	6.5	Feeling swollen	14	44	20.2
Feeling swollen	15	71	10.6	Work/school	15	28	6.2	Skin dry/itchy	15	44	20.2

Referral to psychological care services was discussed with patients based on genetic counselor discretion; these discussions were not limited to patients in the high distress group. Only one patient accepted an offer of referral. Stated reasons for declining referral included having an existing relationship with a psychological care professional, the presence of a strong support system, and/or experiencing predominantly physical rather than emotional concerns.

## Discussion

To date, most studies have focused on utilization of the DT and PL among oncology patients while studies in the hereditary cancer risk setting have primarily focused on comparison of the DT and PL to other psychosocial assessment tools.^7,9^ Our study is the first to our knowledge to examine correlates of distress in a North American population in an outpatient oncology genetics setting. The proportion of patients reporting high distress (approximately one third) is in line with prior studies of DT use in a cancer genetics setting [11]. While only half of our study population had a personal history of cancer, there were no significant differences between distress level based on this variable. Consistent with previous studies in oncology patients, we found younger age and female sex assigned at birth correlated with being in the high distress group and that the most frequently reported PL concerns were worry, fatigue, and sleep disruption; however, these were reported less frequently overall among our patients compared to prior reports of rates in oncology populations [4, 6]. While higher distress has been reported in oncology populations for individuals assigned female sex at birth, psychological distress inequity is a well-described issue, with women reporting more psychological distress than men in the general population. Individuals who identify as male may repress or under-report symptoms of psychological concerns due to stigma around reporting and/or may present with these conditions in a different manner than those who identify as female [12]. Financial concerns were not a commonly reported stressor in our population, unlike in oncology patients, perhaps due to socioeconomic status of Michigan Medicine’s patient population [5]. Additionally, as only half of our study population had a personal history of cancer, our cohort is not directly comparable to prior research in oncology populations.

The data presented in our study serve to further support the conclusions of previous researchers that the DT is successful at identifying patients within the hereditary cancer risk environment who are experiencing high levels of distress [11]. Similar to previous studies, however, differences in reported distress levels were not easily explainable by reported problems, with patients endorsing similar concerns on the PL irrespective of their degree of distress, although at different magnitudes. A measure has not yet been developed which is both consistent with those validated measures currently applied to patients in the oncology setting and can identify those concerns which may be specific to the patient population in the cancer genetics setting. Our findings underscore the utility of the DT in the cancer risk predisposition environment while highlighting the need for measures which can more effectively identify the specific concerns relevant to patients in this setting and may better correlate to varying degrees of distress.

## Study limitations

The DT and PL did not include any items specific to genetics, such as worry about hereditary cancer risk, limiting the ability of patients to highlight these potentially relevant stressors. Further, the population of this research was drawn from a single clinical site and implicitly reflects a subsection of patients who were able to surpass barriers to accessing genetics care. Since data collection concluded, updated versions of the DT and PL have been released, including changes to the PL intended to better address items relevant to whole-person care (Online Material 2); however, none of these changes specifically address hereditary cancer risk [11]. Data collection concluded over 4 years prior to publication as workflows were adjusted due to COVID and data were analyzed after deciding to end the study. Other limitations include a primarily non-Hispanic White population, a convenience time period/sample, the limited timeframe within which patients had to complete the DT and PL, and a study design which did not track the number of patients who were offered referral to psychological care services or the reasons for offering, limiting our ability to accurately report uptake of referrals and reasons for declination. Data were not gathered with regard to patients’ individual cancer diagnoses among those patients who reported a personal history of cancer; therefore, distress could not be compared between patients with different cancer types.

## Clinical implications

The vast majority (97.5%) of patients presenting to clinic completed the DT and PL tools, highlighting the feasibility of using this tool, or a similar assessment, in this setting. Our data support the conclusions of previous researchers that the DT can assist in identifying patients within the hereditary cancer risk environment who are experiencing high levels of distress. However, items on the PL do not address concerns that are unique to this patient population, and a different tool may be useful to elicit specific concerns in those reporting high distress.

## Conclusions

The results of this investigation support the assertion that the DT and PL represent an informative and feasible assessment of psychosocial distress among patients undergoing evaluation for hereditary cancer risk, while also highlighting the inability of causes presented in the PL to fully explain variance between reported levels of distress. Future studies may consider exploring how genetic counselors apply the DT and PL to their practice in instances where patients report a high level of distress. In addition, research to develop a PL or other tool more applicable to the genetic counseling patient population may be considered to elucidate concerns in patients reporting high distress.

## Supplementary Information

Below is the link to the electronic supplementary material.

Supplementary file1 (PDF 278 KB)

## Data Availability

No datasets were generated or analysed during the current study.
