# Supplementary material for: Use of NCCN distress thermometer in cancer genetics patients
Source: Support Care Cancer. 2025 Apr 26;33(5):423. doi: 10.1007/s00520-025-09473-y (PMC12033117; doi:10.1007/s00520-025-09473-y)
Supplement: Supplementary file 1 — Supplementary file1 (PDF 278 KB) [file 520_2025_9473_MOESM1_ESM.pdf]

Article title: **Use of NCCN Distress Thermometer in Cancer Genetics Patients**

Journal name: Journal of Supportive Care

Author names: Aidan M. Kennedy, Andrea M. Murad, Erika S. Koeppe, Michelle B. Riba, Elena M. Stoffel, Michelle F. Jacobs

Affiliation and e-mail address of corresponding author: Michelle F. Jacobs, Rogel Cancer Center and Department of Internal Medicine, University of Michigan, Ann Arbor, MI, USA, mfjac@med.umich.edu

Online Resource 1. 2019 version of Distress Thermometer and Problem List (39 items). Reproduced with permission from the NCCN Clinical Practice Guidelines in Oncology (NCCN Guidelines®) for Distress Management V.3.2019. © 2019 National Comprehensive Cancer Network, Inc. All rights reserved. The NCCN Guidelines® and illustrations herein may not be reproduced in any form for any purpose without the express written permission of NCCN. To view the most recent and complete version of the NCCN Guidelines, go online to NCCN.org. The NCCN Guidelines are a work in progress that may be refined as often as new significant data becomes available. NCCN makes no warranties of any kind whatsoever regarding their content, use or application and disclaims any responsibility for their application or use in any way.

NCCN DISTRESS THERMOMETER

Instructions: Please circle the number (0–10) that best describes how much distress you have been experiencing in the past week including today.

Extreme distress

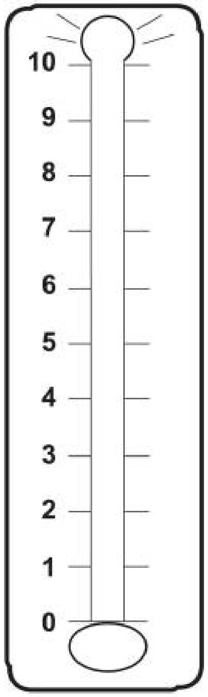

No distress

PROBLEM LIST

Please indicate if any of the following has been a problem for you in the past week including today.
Be sure to check YES or NO for each.

YES	NO	Practical Problems
❑	❑	Child care
❑	❑	Housing
❑	❑	Insurance/financial
❑	❑	Transportation
❑	❑	Work/school
❑	❑	Treatment decisions
		**Family Problems**
❑	❑	Dealing with children
❑	❑	Dealing with partner
❑	❑	Ability to have children
❑	❑	Family health issues
		**Emotional Problems**
❑	❑	Depression
❑	❑	Fears
❑	❑	Nervousness
❑	❑	Sadness
❑	❑	Worry
❑	❑	Loss of interest in usual activities
❑	❑	**Spiritual/religious concerns**

YES	NO	Physical Problems
❑	❑	Appearance
❑	❑	Bathing/dressing
❑	❑	Breathing
❑	❑	Changes in urination
❑	❑	Constipation
❑	❑	Diarrhea
❑	❑	Eating
❑	❑	Fatigue
❑	❑	Feeling swollen
❑	❑	Fevers
❑	❑	Getting around
❑	❑	Indigestion
❑	❑	Memory/concentration
❑	❑	Mouth sores
❑	❑	Nausea
❑	❑	Nose dry/congested
❑	❑	Pain
❑	❑	Sexual
❑	❑	Skin dry/itchy
❑	❑	Sleep
❑	❑	Substance use
❑	❑	Tingling in hands/feet

Other Problems: ______________________________

____

Online Resource 2. 2024 version of Distress Thermometer and Problem List (42 items). Reproduced with permission from the NCCN Clinical Practice Guidelines in Oncology (NCCN Guidelines®) for Distress Management V.2.2024. © 2024 National Comprehensive Cancer Network, Inc. All rights reserved. The NCCN Guidelines® and illustrations herein may not be reproduced in any form for any purpose without the express written permission of NCCN. To view the most recent and complete version of the NCCN Guidelines, go online to NCCN.org. The NCCN Guidelines are a work in progress that may be refined as often as new significant data becomes available. NCCN makes no warranties of any kind whatsoever regarding their content, use or application and disclaims any responsibility for their application or use in any way..

NCCN DISTRESS THERMOMETER

Distress is an unpleasant experience of a mental, physical, social, or spiritual nature. It can affect the way you think, feel, or act. Distress may make it harder to cope with having cancer, its symptoms, or its treatment.

Instructions: Please circle the number (0–10) that best describes how much distress you have been experiencing in the past week, including today.

Extreme distress

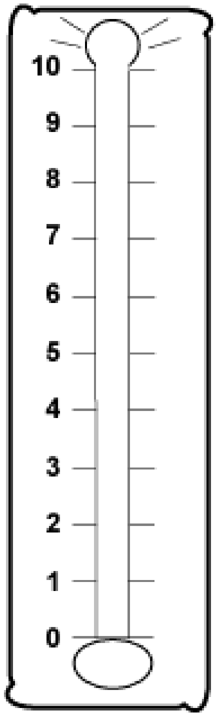

No distress

PROBLEM LIST

Have you had concerns about any of the items below in the past week, including today? (Mark all that apply)

Physical Concerns

- ❑ Pain
- ❑ Sleep
- ❑ Fatigue
- ❑ Tobacco use
- ❑ Substance use
- ❑ Memory or concentration
- ❑ Sexual health
- ❑ Changes in eating
- ❑ Loss or change of physical abilities

Emotional Concerns

- ❑ Worry or anxiety
- ❑ Sadness or depression
- ❑ Loss of interest or enjoyment
- ❑ Grief or loss
- ❑ Fear
- ❑ Loneliness
- ❑ Anger
- ❑ Changes in appearance
- ❑ Feelings of worthlessness or being a burden

Social Concerns

- ❑ Relationship with spouse or partner
- ❑ Relationship with children
- ❑ Relationship with family members
- ❑ Relationship with friends or coworkers
- ❑ Communication with health care team
- ❑ Ability to have children
- ❑ Prejudice or discrimination

Practical Concerns

- ❑ Taking care of myself
- ❑ Taking care of others
- ❑ Work
- ❑ School
- ❑ Housing
- ❑ Finances
- ❑ Insurance
- ❑ Transportation
- ❑ Child care
- ❑ Having enough food
- ❑ Access to medicine
- ❑ Treatment decisions

Spiritual or Religious Concerns

- ❑ Sense of meaning or purpose
- ❑ Changes in faith or beliefs
- ❑ Death, dying, or afterlife
- ❑ Conflict between beliefs and cancer treatments
- ❑ Relationship with the sacred
- ❑ Ritual or dietary needs

Other Concerns:

____

____

____
